# From Molecule to Patient Rehabilitation: The Impact of Transcranial Direct Current Stimulation and Magnetic Stimulation on Stroke—A Narrative Review

**DOI:** 10.1155/2023/5044065

**Published:** 2023-02-28

**Authors:** Anca Badoiu, Smaranda Ioana Mitran, Bogdan Catalin, Tudor Adrian Balseanu, Aurel Popa-Wagner, Florin Liviu Gherghina, Carmen Valeria Albu, Raluca Elena Sandu

**Affiliations:** ^1^Department of Neurology, Clinical Hospital of Neuropsychiatry, 200349 Craiova, Romania; ^2^Department of Physiology, University of Medicine and Pharmacy of Craiova, 200349 Craiova, Romania; ^3^Experimental Research Centre for Normal and Pathological Aging, University of Medicine and Pharmacy of Craiova, 200349 Craiova, Romania; ^4^Department of Physical Medicine and Rehabilitation, University of Medicine and Pharmacy of Craiova, 200349 Craiova, Romania

## Abstract

Stroke is a major health problem worldwide, with numerous health, social, and economic implications for survivors and their families. One simple answer to this problem would be to ensure the best rehabilitation with full social reintegration. As such, a plethora of rehabilitation programs was developed and used by healthcare professionals. Among them, modern techniques such as transcranial magnetic stimulation and transcranial direct current stimulation are being used and seem to bring improvements to poststroke rehabilitation. This success is attributed to their capacity to enhance cellular neuromodulation. This modulation includes the reduction of the inflammatory response, autophagy suppression, antiapoptotic effects, angiogenesis enhancement, alterations in the blood-brain barrier permeability, attenuation of oxidative stress, influence on neurotransmitter metabolism, neurogenesis, and enhanced structural neuroplasticity. The favorable effects have been demonstrated at the cellular level in animal models and are supported by clinical studies. Thus, these methods proved to reduce infarct volumes and to improve motor performance, deglutition, functional independence, and high-order cerebral functions (i.e., aphasia and heminegligence). However, as with every therapeutic method, these techniques can also have limitations. Their regimen of administration, the phase of the stroke at which they are applied, and the patients' characteristics (i.e., genotype and corticospinal integrity) seem to influence the outcome. Thus, no response or even worsening effects were obtained under certain circumstances both in animal stroke model studies and in clinical trials. Overall, weighing up risks and benefits, the new transcranial electrical and magnetic stimulation techniques can represent effective tools with which to improve the patients' recovery after stroke, with minimal to no adverse effects. Here, we discuss their effects and the molecular and cellular events underlying their effects as well as their clinical implications.

## 1. Introduction

Stroke represents one of the main causes of death and a major cause of disability worldwide, with most survivors reporting a decrease in life quality [1, 2]. With an annual increase in its incidence, stroke involves significant economic costs both direct and indirect [3, 4]. As most patients present far beyond the therapeutic window for thrombectomy/thrombolysis, rehabilitation is their only option to improve physical, cognitive, communicative, emotional, and social status [2, 5]. A plethora of rehabilitation programs aimed to improve motor function, balance, walking, and daily living activities have been developed and are being used by healthcare professionals. For example, thousands of repetitions of reach-to-grasp movements are necessary to have an impact on the functional recovery of the upper limb after stroke [6]. Specific recovery strategies seem to work better than others depending mainly on the extent of the infarct area. As such, patients who do not suffer a visual impairment can be subjected to movement performance therapies using mirrors, video, or graphical representations of three-dimensional motion capture as feedback [7, 8]. The major aim of any physical therapy is to promote neuroplasticity and motor recovery after a stroke [9]. The amount and intensity of exercise, personal implication and/or determination, and task-oriented training play a crucial role in the outcome [10]. However, physical recovery is highly dependent on the severity of the stroke. A severe stroke (significant brain tissue damage) induces multiple neurological impairments leading to a considerable loss of function [11]. For those patients, rehabilitation is particularly focused on both function restoration (not always possible or often incomplete) and reduction of immobility-related complications, a burden for caregivers of severe stroke survivors.

With classical rehabilitation having certain limitations in severe cases of stroke, modern techniques such as transcranial magnetic stimulation (TMS) and transcranial direct current stimulation (tDCS) started to make their way as an alternative or complementary method in impacting the consequences of stroke. Being relatively inexpensive and easy to administer [12], in recent years, these noninvasive brain stimulation (NIBS) techniques were applied for the treatment of a variety of conditions in different specialties such as psychiatry, neurology, and rehabilitation. In poststroke rehabilitation, they seem to bring improvements mainly through a cellular process of neuromodulation [13–15], as they counteract the molecular and cellular mechanisms involved in the pathophysiology of cerebral ischemia [16–20]. These NIBS techniques exert their neuroprotective [19–22] or neuroregenerative [23–26] characteristics principally by modifying brain excitability [18, 27–29]. However, their effects were not always favorable, and they seem to be influenced by the type of protocol used [21, 28] or by the heterogenous capacity of individuals to induce M1 plasticity, both in healthy and poststroke-treated patients [30–32]. Regarding protocols, a meta-analysis on 445 stroke patients evidenced that bilateral transcranial electric stimulation and cathodal tDCS over the contralesional hemisphere were superior to other stimulation montages/patterns/protocols [33]. Promising results were also obtained with different protocols of NIBS applied to poststroke survivors. A meta-analysis of more than 600 subacute and chronic poststroke patients revealed the beneficial effects of combined TMS and mirror therapy and tDCS and mirror therapy on upper extremity dysfunction [34].

The moment at which NIBS is applied after stroke affects patients' recovery. For example, encouraging results were observed after repetitive tDCS, with amelioration of the motor and somatosensory functions in patients during the first-month poststroke [35]. Interestingly, a similar positive outcome was also reported for chronic patients [34, 36, 37] or even severely ill patients [38]. Clear benefits were observed on motor function with TMS being applied during the acute phase of stroke [39–41], while more diverging results were obtained in the subacute or chronic phases by using only NIBS [42–44]. Another beneficial result of NIBS on poststroke patients is the reduction in depression scale scores [45–47] and improvement of aphasia [48], episodic memory, working memory [49], or attention [50, 51].

In this review, we will focus on reported experimental and clinical findings underlying the molecular, cellular, and clinical reasoning behind modern poststroke rehabilitation strategies. The present work reflects both our own experience and literature search online using resources from PubMed, Clarivate, and other scientific databases.

## 2. Transcranial Electric Stimulation Overview

Transcranial electric stimulation (TES) is a noninvasive method used to modulate brain functions (i.e., motor, sensory, and cognitive) with applicability to many neurological conditions such as stroke [52], multiple sclerosis [53, 54], epilepsy [55], Alzheimer's disease [56, 57], and Parkinson's disease [58, 59]. It uses scalp electrodes to deliver positive (cathodal) or negative (anodal) currents to specific cortical regions. The low intensity of the current (1-2 mA) does not trigger an action potential but rather alters neuronal excitability by modifying the membrane polarization [18]. Anodal stimulation generates depolarization, while cathodal stimulation results in hyperpolarization [27, 60]. The main effect of TES can be the modulation of ongoing neural oscillations [61, 62] or neuroplasticity induction [63, 64]. Thus, the neurophysiological effects of TES can be classified as immediate [65] and long-lasting [66]. While immediate effects are due to changes in synaptic activity level and neuronal membrane properties [62, 65], long-lasting effects outlast the period of stimulation and are generated through modifications of intracellular calcium dynamics and mechanisms of synaptic plasticity supporting long-term potentiation (LTP) or long-term depression (LTD) [63, 64].

In practice, three different approaches to TES are known: transcranial direct stimulation (tDCS) [18, 67], transcranial alternating current stimulation (tACS) [68, 69], and transcranial random noise stimulation (tRNS) [70]. The main difference between these three approaches lies in the way the current is delivered. In tDCS, the electrical current flows unidirectionally from the anode to the cathode. In tACS, the current flows sinusoidally with a particular frequency and stimulation amplitude from the anode to the cathode in one half-cycle and in the reverse direction in the second half-cycle. In tRNS, alternating current oscillates at random frequencies [71, 72]. Generally, two protocols of tDCS are used for the treatment of stroke. Unilateral tDCS involves the placement of an active electrode, either anodal or cathodal, over the brain area (i.e., primary motor cortex-M1) with a contralateral cathodal or anodal supraorbital reference electrode. Dual tDCS is a technique used by placing both electrodes simultaneously over the hemispheres: the cathode is placed over the M1 of the nonlesioned hemisphere, and the anode is placed over the M1 of the lesioned hemisphere [73].

### 2.1. Experimental Data Supporting the Therapeutic Use of TES

Studies done on animal stroke models showed that TES provides *neuroprotection* [17, 21, 22, 74–76] by attenuating some of the ischemia-induced cerebral injury mechanisms such as glutamate excitotoxicity [77–80], neuroinflammation [81–83], oxidative stress [84–86], blood-brain barrier dysfunction [87, 88], apoptosis [89, 90], autophagy [91–94], and cortical spreading depression [95, 96]. In the subacute and chronic phases of ischemic stroke, the *neuroregenerative* effects of this noninvasive brain stimulation [14, 23, 62, 97, 98] are more prominent and most likely reflect enhancement of neurogenesis [24, 99], synaptogenesis [100, 101], angiogenesis [17], and neurotransmitter metabolism [102–104]. In many clinical studies, acute and long-term treatment with TES is proved safe and effective in improving functional outcomes [36, 105–107].

#### 2.1.1. Cerebral Molecular Response to TES

The exact molecular mechanism by which TES exhibits beneficial effects in poststroke patients is still largely unknown (Table 1). Mounting evidence shows that, most likely, TES does not have a singular effect that stimulates recovery but rather influences many processes such as astrocytic calcium and glutamate pathways [108] and reduced the number NMDA receptor 1 (NMDAR1) in the hippocampus [109] resulting in a decrease spontaneously of *peri-infarct depolarization* (PID). The direct consequence of all the molecular effects adds to two main effects. The first is that cathodal tDCS (C-tDCS) decreases the DNA fragmentation and lowers the number of Bax- and caspase-3-positive cells, with a simultaneous increase in Bcl-2 protein expression and Bcl-2/Bax ratio, both reliable markers for the *antiapoptotic pathways* [109]. The second is that C-tDCS lowers the expression of stress proteins and suppresses global protein synthesis, thereby providing neuroprotection [22, 110] by reducing neuronal activity, and thus, it decreases cell metabolism, thereby providing neuroprotection [109] and promoting cell survival after an ischemic lesion. The main molecular pathway involved in in this process is inhibition of caspase-3-dependent apoptosis that seems to be promoted by TES-dependent activation of brain-derived neurotrophic factor (BDNF) and phosphoinositide 3-kinase (PI3K)/Akt/mammalian target of rapamycin (mTOR) pathway [17, 111, 112]. Molecular markers of inflammation, such as hippocampal levels of IL-1b and TNF-a, were found to be decreased, after C-tDCS, in MCAO mice [109]. Likewise, rodents subjected to C-tDCS or A-tDCS had increased levels of superoxide dismutase (SOD) and decreased malondialdehyde (MDA - a membrane lipid peroxidation marker), thus attenuating the *oxidative stress* induced by cerebral ischemia [109].

The consequences of electric stimulation (ES) on the molecular mechanisms also come with functional changes. One of the most interesting observations was the change in membrane polarity induced by direct current stimulation (DCS), which, in turn, modulates Ca^2+^ influx through activation or inhibition of NMDA receptors [102]. This modulation can activate then the enzyme cascades that add or remove glutamatergic AMPA receptors on the postsynaptic membrane, thus strengthening or weakening synaptic connections [113]. The capacity of DCS to influence the strength of neuronal connections has a direct effect on LTP. *In vitro* experiments done on brain slices investigating the connection strength between pyramidal cells of the CA3 hippocampal region and neurons of the CA1 area were able to show that anodal DCS markedly increases LTP, whereas cathodal DCS reduces it. These effects are most likely explained by the increase in Zif268- and C-fos protein-positive cells found in the CA subregions after anodal and cathodal stimulation [114]. Apart from the above-mentioned neuroplastic effects, the GABAergic system seems to play a role in tDCS-induced plasticity. Simultaneous administration of lorazepam (a GABA receptor agonist) to healthy subjects caused a reduction in neuroplastic excitability induced by anodal tDCS in the early phase and an enhancement of it in the late phase [103]. As for synaptic plasticity mediated by BDNF, *in vivo* studies support its enhancement by DCS [115, 116], while an *in vitro* experiment showed the opposite effect [114]. Neuroprotection following ES can be enhanced through the *suppression of autophagy*, another damaging effect excessively triggered by acute and severe cerebral ischemia [117]. The reperfused rat somatosensory cortex that was subjected to ES showed an upregulation of P62 coupled with the suppression of LC32, two apoptotic markers that vary according to the autophagic flux. [111].

At a molecular level, an early A-tDCS application was shown to increase the expression of microtubule-associated protein 2 (MAP-2) and growth-associated protein 43 (GAP-43). This increase directly impacts dendritic plasticity, axonal regrowth, and synaptogenesis both in the ischemic penumbra and in the contralateral cortex with a measurable functional recovery assessed by (improved Barnes maze performance, motor behavioral index scores, and beam balance test) [101]. After global ischemia, A-tDCS increased the expression of postsynaptic density protein 95 and synaptophysin both in the cortex and hippocampus with beneficial effects on recovery assessed by quantitative electroencephalogram, neurological deficit score, and 96 h survival [118].

#### 2.1.2. Cerebral Cellular Response to TES

**Figure 1:**
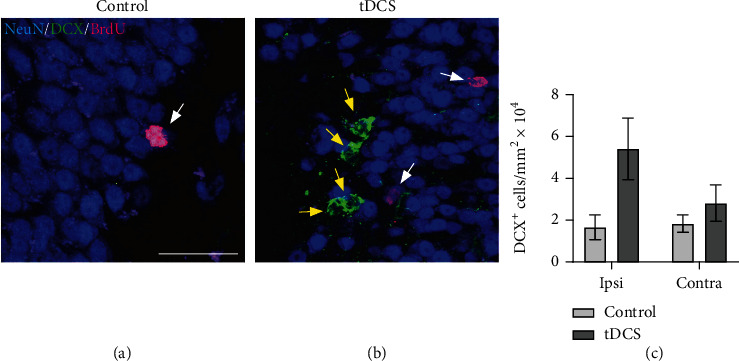
Increase of hippocampus neurogenesis in poststroke mice 14 days after receiving tDCS. (a) Compared to controls, in ES rats, we were able to identify a hippocampal increase in the number of (b) DCX (yellow arrows) and BrdU cells (white arrows). (c) This effect was seen in both the ipsilateral (Ipsi) and contralateral (Contra) hippocampus.

It is not surprising that all molecular changes following ES will also elicit a cellular response. One of the most important cellular consequences of TES is an increase in *neurogenesis* both in healthy [23] and injured central nervous system [24, 99, 119]. Research data showed that TES (subconvulsive train of 30 mA, 60 pulses/sec, 0.5 ms pulse width, 1 s duration, and in total for 5 s) during the subacute phase of stroke was followed by an increase in the number of BrdU^+^/tubulin beta III^+^ cells in the infarct core. The same stimulation elicited increased subventricular (SVZ) ratio of BrdU/DCX^+^ cells and an increase in the number of ipsilateral hippocampus neurons positive for doublecortin (DCX) [24] (Figure 1). Other studies showed that MACO rats subjected to C-tDCS (500 *μ*A, 15 minutes, once per day for 5 days in the acute and 5 days in the subacute phase) were able to evoke an increase in the Nestin^+^/Ki67^+^ and Ng2^+^ cells in the SVZ [99] while A-tDCS increased number of DCX^+^ cells in the SVZ, 10 days after stroke [119].

The generation of new brain cells after TES may explain the increase in its structural, functional, and connective reorganization. Increased structural *neuroplasticity* after stroke, evaluated by the density of dendritic spines in the mouse cerebral cortex, was reported after daily sessions of A-tDCS over the ipsilesional motor cortex paired with C-tDCS stimulation of the contralesional motor cortex. Significant improvement in motor function, assessed by beam walking test scores, was observed in the tDCS group compared to the MCAO group [100].

After alteration of inflammatory molecular pathways, MCAO receiving mouse C-tDCS showed reduced levels of macrophage activation markers (CD68^+^ cells), microglia (Iba1^+^ cells), lower astrogliosis (GFAP^+^ cells), less neutrophils (MPO^+^), and mononuclear cells (CD45^+^) in the ischemic penumbra of the cerebral cortex [17, 21, 111]. Interestingly, MCAO mice that received anodal tDCS (A-tDCS) treatment had an increase in CD45^+^ and MPO^+^ cells around the ischemic cortex and in the striatum [21].

An extensive cerebral vasculature is necessary for the support of these neuroprotective and neuroregenerative effects. Thus, TES was shown to enhance *angiogenesis* in animal stroke models through the increased number of laminin-positive vessels in the ischemic penumbra and upregulation of vascular endothelial growth factor (VEGF) [17]. On the other hand, the effects of TES on the postischemic *blood-brain barrier (BBB)* are conflicting. Thus, anodal stimulation amplified the BBB damage with a subsequent increase in edema and ischemic lesion volume probably caused by the accumulation of endogenous IgG in the ipsilateral ischemic hemisphere compared to the contralateral healthy hemisphere and the significant disruption of blood vessel tight junctions [21]. Also, in healthy rat, the brain stimulated with A-tDCS transiently enhanced the permeability of the BBB through activation of nitric oxide synthase, disruption of the endothelial glycocalyx, basement membrane, and the tight junctions, as well as the increase of the gap width between endothelial cells and basement membrane [120]. Some of these effects were also found in an *in vitro* study [121]. However, C-tDCS applied to stroke rat models reduced the ischemic volume, brain edema [21], and nitric oxide synthase level [109]. The integrity of tight junctions after C-tDCS was similar to that of nonstimulated animals, but the IgG leakage was lower compared to both the sham and A-tDCS groups [21].

### 2.2. Clinical Studies Using TES

Several clinical studies investigated the effects of tDCS on motor recovery in stroke patients (Table 2). In a pilot randomized controlled trial, a current of 1.5 mA or sham current was delivered for 20 minutes hourly over a period of 6 hours and 20 minutes in *hyperacute* middle cerebral artery territory stroke patients receiving reperfusion therapy (intravenous thrombolysis alone or mechanical thrombectomy with or without prior intravenous thrombolysis). Although no major adverse effects (death or neurological deterioration) were reported, the study found no difference between the treated and sham groups. Although the results were not satisfactory, patients receiving reperfusion therapy and ES had smaller infarct volumes. The potential benefits of C-tDCS in patients were also shown for patients with a National Institute of Health Stroke Scale (NIHSS) score of >10 or large vessel occlusion who showed improved functional independence at 3-month poststroke [107]. The lack of a clear benefit in this study was attributed to the fact that on a molecular level, only certain C-tDCS protocols can reduce PID and influence local neuronal networks [107, 122]. On the other hand, C-tDCS exerts its inhibitory effects depending on the organization of cortical neuronal arrangement (i.e., lissencephaly and gyrencephaly) [65]. Building on the partial success of these findings, two clinical trials, TESSERACT and TESSERACT-BA, were approved. The TESSERACT study is testing the use of incremental C-tDCS doses in patients ineligible for reperfusion therapies (URL: https://www.clinicaltrials.gov and unique identifier: NCT03574038) while the TESSERACT-BA study is investigating tDCS in acute stroke patients with substantial salvageable penumbra due to a large vessel occlusion before and after endovascular therapy (URL: https://www.clinicaltrials.gov and unique identifier: NCT04061577). No data had been reported prior to the writing of this review.

#### 2.2.1. TES Influence on the Outcome of Acute and Subacute Stroke

The use of tDCS was not duplicated in other small clinical trials. Bihemispheric tDCS modulation in *acute stroke patients* (48-96 h after stroke) for five continuous days, 40 minutes per day over the primary motor cortex (M1), did not show any clinical benefit beyond the one achieved by the physical therapy alone or through spontaneous recovery [123]. However, some neurophysiological changes were noted (i.e., decrease of the interhemispheric imbalance of excitability and modulation of plasticity), but the lack of clinical improvement was most likely caused by the inappropriate inhibition of the unaffected hemisphere through A-tDCS during the acute stage of stroke [124], as well as the unsuitable tDCS parameters of stimulation [125]. Similarly, A-tDCS applied over the affected motor cortex of acute stroke patients (2 mA for 20 min daily for five consecutive days) showed no significant improvement in NIHSS and Fugl-Meyer scores compared to sham. The lack of efficacy was also attributed to the imbalance of excitability generated through this technique [126].

One targeted study investigated the potential of A-tDCS to improve dysphagia in acute-subacute stroke patients with unilateral ischemic infarction. It is reported that ES sessions (2 mA either twice daily for a total of 20 minutes or alternating with sham stimulation daily for a total of 20 minutes) performed along with standardized swallowing over five days, starting from day 2 to 6 after stroke onset, did not decrease aspiration risk assessed through Penetration and Aspiration Scale score. Since A-tDCS exerts its effects mainly by modulation of activity in the other intact hemisphere, the limitations, in this case, were most likely due to the extent of damage to the corticobulbar tracts in each case [127].

The use of tDCS on stroke patients during the *subacute period* elicits diverging results. Six consecutive sessions (25 minutes at 2 mA daily) of either C-tDCS over the unaffected hemisphere or A-tDCS over the affected hemisphere administered in early subacute stroke patients seem to have clinical improvements both in the upper and lower limb only after 6 sessions [106]. However, a stimulation of 2 mA using A-tDCS or C-tDCS combined with robot-assisted bilateral arm training applied to subacute stroke patients (3 to 8 weeks from stroke onset) every workday for 6 weeks did not have any additional effect compared to sham [128].

Although some minimal effects were reported, the general consensus seems to be that DCS has a minimal impact on acute and subacute stroke patients. This low efficacy could be explained in several ways. First, a lack of standardization in the way tDCS is applied in stroke patients and the optimal timing of ES. Also, the current characteristics are still unknown. Second, it could be that cellular effects seen in rodent studies have only a limited impact on large lesions or the effect is difficult to quantify in a clinical setting. Whatever the case, the results reported by various clinical studies suggest that some benefits exist and an improvement may be possible.

#### 2.2.2. TES Influence on the Outcome of Chronic Stroke

While the acute and subacute effects of tDCS are still debated, the benefits of tDCS stimulation are far more obvious in *chronic stroke patients*. Several studies showed motor improvement after A-tDCS, especially in association with other recovery strategies. Thus, A-tDCS (1 mA for 20 min on the affected hemisphere) given at 1.9 to 8.9 years after stroke, preceding motor therapy of the upper limb, was able to evoke a significant functional improvement of the paretic hand as measured using the Jebsen–Taylor Hand Function Test compared with motor therapy alone. Furthermore, this effect outlasted the stimulation period [105]. Even patients suffering a stroke up to 7.2 years prior to combined peripheral nerve stimulation of the paretic hand (5 single pulses of 1 ms duration delivered at 10 Hz applied simultaneously over the median and ulnar nerve at the wrist) and A-tDCS applied over the ipsilesional primary motor cortex at an intensity of 1 mA for 20 min showed an improved motor performance, evaluated through the number of correct key presses on a special keyboard containing only 5 keys as compared with motor practice or with practice combined with either intervention alone. This study also reported that the effect outlasted the stimulation and training [129]. Bihemispheric tDCS at 1.5 mA for either 20 min (current density 0.06 mA/cm^2^) or 30 min repeated in five sessions done on patients that were subjected to physical/occupational therapy also showed improvements in motor function in chronic stroke patients. The effects were assessed by either Upper Extremity Fugl-Meyer, Wolf Motor Function Test, or Motor Assessment Scale, Tardieu Scale, and grip strength [36, 130].

## 3. Transcranial Magnetic Stimulation—Short Introduction

Transcranial magnetic stimulation is another noninvasive technique that is able to modulate brain activity already used in different clinical settings. For example, TMS is used as a treatment method for several psychiatric pathologies such as depression [131, 132] or schizophrenia [133]. It is also applied in some neurologic pathologies to improve the outcome of different movement disorders [134, 135], stroke [105], multiple sclerosis [136], Alzheimer's disease [137], and disorders of consciousness [138]. Extensive recent reviews of repetitive TMS on specific poststroke consequences, such as poststroke dementia [139] and poststroke depression [140], have been made, and on the matter, we encourage their reading for further in-depth knowledge.

As is the case with ES, in practice, TMS can also be applied under different protocols. Depending on the applied protocol, TMS can have different effects on brain excitability. Thus, high-frequency repetitive TMS (HF-rTMS) (>1 Hz) [28] and intermittent theta burst stimulation (iTBS) [29] can increase cortical excitability, while TMS protocols using low-frequency repetitive TMS (LF-rTMS) (≤1 Hz) [141] or continuous theta burst stimulation (cTBS) [29] decrease it. One major advantage of using TMS is that its effects last beyond the stimulation period [142]. However, for accurate and meaningful interpretation of TMS results, controls need to be matched at least for age, height, and sex [143].

### 3.1. Experimental Data Supporting the Use of TMS in Stroke

The molecular and cellular mechanisms through which TMS exerts its effects on stroke are not fully elucidated (Table 3). Although technically TMS is more difficult to use in an experimental setup, especially in rodents that have a small cortical volume, several studies were able to show changes in molecular and cellular responses.

#### 3.1.1. Cerebral Molecular Response to TMS

Extensive molecular research found that cTBS reduces poststroke *neuroinflammation* by lowering the levels of cytokines associated with infiltrating immune cells into the central nervous system (i.e., CNTF, CX3CL1, IFN-r, IL-*α*, IL-1*β*, IL-1ra, IL-2, IL-3, IL-6, IL17, and TNF*α*) or the cytokines related to endothelial inflammation (i.e., CD54, CXCL9, CXCL10, and CCL5) [19, 20]. Adding to this anti-inflammatory effect is the attenuation of *oxidative stress* by reducing the NADPH oxidase activity with subsequent reduction in MDA and 4-hydroxynonenal (another marker of lipid peroxidation) and increasing manganese-superoxide dismutase which clears the free radicals generated by mitochondrial respiration [20, 144, 145].

However, one of the most important molecular effects of TMS is its influence of *neurotransmitter metabolism*. Post TMS, different neurotransmitter levels increase or decrease depending on the investigated region. For example, TBS lowered the level of cerebellar glutamate in the extracellular space of healthy rodents by increasing its uptake from the synaptic cleft and its turnover in neurons. This is done by increasing the number of plasmatic glutamate transporter 1 and by lowering the levels of vesicular glutamate transporter 1 [146]. However, LF-rTMS could not evoke any change in glutamate and glutamine levels in the primary motor cortex but increased GABA levels [147]. Increased extracellular dopamine and glutamate levels in the nucleus accumbens were also found after TMS [148]. Either this heterogeneous response is due to a random way the brain responds to TMS or it can be attributed to the variation in the applied techniques used in animal models. HF-rTMS applied in the subacute phase of stroke in a rat model did not found changes in the expression of NMDA and MAP-2 around the peri-ischemic area, questioning the role of TMS in synaptic plasticity, LTP, and dendritic plasticity in the early phases of stroke. Although no evidence of neuroplasticity was reported, the same groups showed that the animals receiving HF-rTMS had an improvement in functional recovery [149].

The potential of TMS to alter *apoptosis/augmented autophagy* might be of great importance in the clinical practice, as preventing additional cellular death after stroke generates more recovery potential compared to neuronogenesis or anti-inflammatory strategies. In a rat model, HF-rTMS applied during the acute and subacute phase of cerebral ischemia inhibited *apoptosis* by significantly enhancing the expression of Bcl-2 and reducing the expression of Bax compared to controls [149, 150]. The use of c-TBS was reported to have an inhibitory effect on the activation of caspase-3 and caspase-9 [20], while rTMS can increase the ratio of LC3-II/I and decrease p62 through NMDAR–Ca^2+^–mTOR signaling [151]. Although this TMS effect can be important for poststroke recovery, it is not completely clear if this potentially augmented autophagy of TMS is eliciting a beneficial effect through clearance of the postischemic debris rather than prevention of neuronal death.

Recent reports showed that TMS can influence the integrity of the BBB and promote *angiogenesis.* The observation was done by using rTMS on a rat photothrombotic stroke model. Stimulated animals had less ischemic-induced degeneration and showed an upregulation in important BBB components such as zona occludens-1, claudin-5, occludin, and caveolin-1. In addition, a reduction in the extravasation of IgG into the peri-infarcted area and upregulation of Col IV, an essential element to vascular structure, were also reported [19]. An increase in angiogenesis-related proteins, such as matrix metalloproteinase-9 and VEGF plus the colocalization of vascular endothelial with cellular proliferation markers RECA1/Ki67 and CD31/BrdU, suggests the angiogenic potential of TMS [19].

#### 3.1.2. Cerebral Cellular Response to TMS

After TMS, a plethora of other cellular phenomena has been reported, especially in animal models of stroke. Apart from this molecular effect, cTBS has cellular anti-inflammatory effects as evidenced by decreasing the number of Iba1^+^ and GFAP^+^ cells in the peri-infarct region [20]. In the early phase of stroke, both HF-rTMS and iTBS increased the number of Ki67 and DCX/Nestin or NeuN^+^ cells suggesting that they could promote an increase in the neural stem cells (NSC) followed by a migration to the peri-infarct striatum. Furthermore, by analyzing the SVZ number of Ki67^+^, an increase was observed after rTMS [152]. IN the subgranular zone, an increase in the ratio BrdU/Nestin^+^ cells was observed after rTMS in MCAO rats [150]. HF-rTMS applied over the primary motor cortex in healthy mice modulates spinogenesis by increasing the number and complexity of thin spines in apical and basal dendrites [25], showing that it can also have a *neuroplastic effect*.

By combining peripheral nerve stimulation and TMS application, an increase in the expression of MAP-2 and GAP-43 in the ischemic penumbra was reported in the acute phase focal cerebral ischemia and reperfusion, suggesting that TMS is promoting dendritic plasticity and axonal regrowth [153]. The same association was shown to also promote functional neuroplasticity by enhancing LTP at synapses in the CA3 and CA1 regions of the hippocampus through upregulation of mRNA expression of BDNF and NMDAR1, with subsequent amelioration of poststroke impaired learning and memory [154]. If the effects on NMDA-mediated neuroplasticity are paradoxical, neuroplasticity mediated through upregulation of c-Fos and BDNF expressions was supported in a study that applied LF-rTMS in the early phase of stroke in rodents, leading to a neurological function recovery [26]. All this data suggests that the effect of TMS on poststroke recovery might be the overall result of an accumulation of different activity-dependent synaptic plasticity, also known as metaplasticity [155].

### 3.2. Clinical Studies Using TMS

With the successful use of TMS in other clinical settings [131, 132] and considering the molecular and cellular results from animal models, it did not take long until TMS was tested on stroke patients (Table 4).

#### 3.2.1. TMS Influence on the Outcome of Acute and Subacute Stroke

Compared to DCS, data generated from patients receiving TMS in the *acute period of stroke* was shown to have beneficial effects on *motor function*. One of the first studies used a combination of standard physical rehabilitation strategies (passive limb manipulation from the second day, increasing, by the end of the first week, to more active movements if patients improved function), medical therapies (anticoagulants, antiplatelets, and nootropics), and HF-rTMS (10-second trains of 3 Hz stimulation with 50 seconds between each train for 10 days) over the stroke hemisphere of early postischemic patients. The study reported improvement in clinical scales (Scandinavian Stroke Scale, NIHSS, and Barthel scores) in patients receiving magnetic stimulation [39], suggesting that TMS could impact specific motor impairments. Due to the nature of TMS, specific cortical areas can be targeted, as such, by applying HF-rTMS over the oesophageal cortical area of the affected hemisphere (10 trains of 3 Hz stimulation, 10 min every day for five consecutive days), a clinical improvement of patients suffering from *poststroke dysphagia* assessed through Dysphagia Outcome and Severity scale was observed. The observed effect was speculated to be a consequence of the increase in corticobulbar projection excitability of both hemispheres [40]. Other LF-rTMS protocols in *subacute stroke patients* also reported encouraging results [156].

With the large motor deficit, capsular stroke patients have generally a poor recovery prognostic [157]. Interesting one of the fists reports that used TMS on capsulat patients utilized two distinct protocols: one using iTBS on the affected side for a total of 600 pulses at an intensity of 80% resting motor threshold (RMT) for 10 days and the other using LF-rTMS on the unaffected hemisphere for a total of 1200 pulses at an intensity of 110% RMT for 10 days. After the two, an enhanced movement and reduced spasticity of the affected limbs compared to the sham group was observed. The study reported improvement in clinical indicators such as Fugl-Meyer Assessment, Stroke Impairment Assessment Set, finger-function test, grip strength, and increase in motor evoked potential amplitude, measured in the first dorsal interosseous on the affected side [41]. However, it should be noted that the results are significant only compared to shams.

The complexity of poststroke disabilities does not restrict to only the motor ones. As such, TMS was used to investigate other nonmotor outcomes. Using a 1 Hz for five minutes with 90% RMT, performed four times, for a total of 1,200 stimulation events, for four weeks, five times each week and 10 minutes each day, LF-rTMS was reported to ameliorate higher-order cerebral functions such as unilateral spatial neglect as assessed by Line Bisection Test and Albert Test [42]. Similarly, 5 days per week for 2 weeks, 20 minutes each day, was shown to improve aphasia (measured by the Aachen Aphasia Test) [158], and even visuospatial neglect (evaluated through Behavioral Inattention Test) was reposted to be impacted by cTBS [159]. Other partial benefits were reported after using different HF-rTMS protocols such as improved the motor function of the affected upper limb, but not the lower one [160].

#### 3.2.2. TMS Influence on the Outcome of Chronic Stroke

While acute and subacute results after TMS are generally encouraging, depending on the type of used protocol, different groups reported diverging recovery outcomes of *chronic stroke patients.* While bilateral TMS using 1 Hz and 50 sec train duration over the unaffected hemisphere, alternating with 10 Hz and 5 sec train duration over the affected hemisphere, with an interval of 5 sec for 20 times and LF-rTMS (1 Hz, 90% RMT, 25 min) applied to these groups of patients was reported to enhance motor skill acquisition in paretic hand movement evaluated through acceleration and pinch force [44, 161], the use of HF-rTMS had less than expected effects when applied to the affected cortex. Further diverging results were reported, with one study (with a protocol of 20 pulses at 10 Hz, 80% RMT, for a total of 160 pulses, in 2 sessions) showing improvements in hand motor performance assessed through movement accuracy and movement time [162], while another (90% RMT, 10 Hz, 1000 stimuli) showed no effect on motor function [161]; however, in this case, there was a lack of a stereotactic system with integrated MRI data or insufficient stimulation power to increase cortical excitability [161].

TMS was also used in an attempt to improve other aspects of poststroke recovery. In chronic poststroke dysphagia, HF-rTMS (10 sessions of rTMS at 3 Hz applied to the pharyngeal motor cortex bilaterally), followed by 20 min of intensive swallowing rehabilitation exercise, improved laryngeal elevation delay time in 4 poststroke patients [163]. The effect was confirmed in a larger study that demonstrated that 500 pulses of 10 Hz rTMS over the ipsilesional and 500 pulses of 10 Hz rTMS over the contralesional motor administered daily for 2 consecutive weeks over the cortical areas projecting to the mylohyoid muscles are effective as an additional treatment strategy to traditional dysphagia therapies, with improvements in Clinical Dysphagia Scale, Dysphagia Outcome and Severity Scale, Penetration Aspiration Scale, and Videofluoroscopic Dysphagia Scale [164]. iTBS (bursts of three pulses at 50 Hz given every 200 milliseconds in two-second trains, repeated every 10 seconds over 200 seconds for a total of 600 pulses) applied to chronic left middle cerebral artery stroke patients with moderate aphasia (≥12 months prior to study participation) proved to be effective clinically, paraclinically, and subjectively. Thus, after rTMS, patients showed improvements in semantic fluency, being able to generate more appropriate words when prompted with a semantic category. fMRI mapping of post-rTMS showed shifts in activations predominantly of the left hemispheric head regions (fronto-temporo-parietal language networks). Also, patients noted a subjective improvement in the Communicative Activity Log [165].

## 4. Conclusions

With the increase in the global aged population, an increase in the incidence of stroke is expected. This will become a larger and larger problem as the number of patients increases more compared to the number of healthcare professionals properly trained to deal with such cases. Therefore, other ways to improve patient outcomes are needed. The results of some clinical studies using TMS in acute and subacute stroke patients paired with the ones from DCS applied to chronic patients could be the aid that stroke patients need, to ensure better results of classical medical recovery and, as such, diminish their disability.

## Figures and Tables

**Table 1:** Main neuroprotective and neuroregenerative effects of transcranial electric stimulation in experimental research.

Model	Stroke stage	Technique	Types of protocol	Cortical effects	Neurogenesis	Neuroprotection	Neural plasticity	Neuroinflammation	Angiogenesis	Oxidative stress	Neurotransmitter metabolism	BBB permeability	Clinical results	Possible signalling pathway	Data
Healthy animals		C-tDCS or A-tDCS	Continuous administration of C-tDCS or A-tDCS for 15 min at 500 *μ*A using a constant current stimulator to a charge density of 128.571 C/m^2^, daily, for 5 consecutive days, followed by a tDCS-free interval of 3 days and another 5 days of electrical stimulation for only half of the animals	Inflammatory modulation through IBA1+ cells, increased ICAM1+ and BrdU+ cells	P	P		P	P				Not analysed	**—**	Rueger et al., [23]
			1 mA current, for 15 min, the inter-tDCS interval longer than 2 h. At the onset and offset of stimulation, the current was slowly ramped up and ramped down over ∼15 s to avoid sudden current change. S-, A-, and C-tDCS were performed in order and repeated for six cycles in each cat	A- and C-tDCS can selectively affect GABAergic and glutamatergic transmissions by reducing GABA and glutamate synthesis		P					P		Reduced glutamate excitotoxicity; A- and C-tDCS can, respectively, enhance and suppress neuronal excitability	Selectively affect GABAergic and glutamatergic transmissions	Zhao et al., [161]
		A-tDCS	3 different doses of weak direct current (0.1, 0.5, and 1 mA) for 20 min (including 30 s ramp up and 30 s ramp down), current density for each dosage being 0.8, 4.0, and 8.0 mA/cm^2^	Enhanced BBB dysfunction (transiently); could be used as a convenient, noninvasive, and selective approach for systemic drug delivery to the central nervous system via the BBB								P	BBB permeability modulation	Temporarily disrupting the structural components forming the paracellular pathway of the BBB	Shin DW et al., [114]

MCAO model	Acute/chronic	t-DCS	Subconvulsive train, 30 mA, 60 pulses/sec, 0.5 ms pulse width, 1 s duration and in total for 5 s at 7 and 24 days after stroke	Possibly reduced glutamate excitotoxicity (significantly downregulated genes Gria 3-glutamate receptor) increased the number of BrdU-labeled tubulin beta III cells in the infarct core of ES animals over controls	P	P							Significant beneficial effect on spatial long-term memory, no beneficial effect on complex sensorimotor skills, detrimental effect on the asymmetric sensorimotor deficit	Possibly AKT/mTOR and *β*-catenin signaling pathways	Balseanu et al., [24], Liu et al., [50]
	Acute		Continuous stimulation for 3 days or 1 week, with square-wave pulses at the duration of 1 ms constant current, with different electric current (0, 100, 200 *μ*A) and frequency (0, 2, 10, 50 Hz). After the 1-week stimulation, the electric stimulation was discontinued	Phosphorylated Akt upregulation of BDNF, GDNF, and VEGF		P		P	P				Ameliorated behavioral impairment; reduced infarct volumes; increased cerebral blood flow through angiogenesis	Stimulation of PI3K/Akt/mTOR pathway	Baba et al., [17]
			Electric current of 20 Hz, 2 ms square biphasic pulse, 100 *μ*A for 30 min, starting at 30 min after reperfusion	Inhibits proliferation and activation of microglia and astrocyte upregulation of BDNF		P		P					Attenuated infarction volume and improved functional recovery; neuroprotection	Stimulation of PI3K/Akt/mTOR pathway, autophagy P62-LC3B-related pathway	Wang et al., [166]
		A-tDCS	Early tDCS, 1 day after ischemia for 5 days and late tDCS, 1 week after ischemia for 5 days	Enhanced levels of MAP-2 and GAP-43 for dendritic and axonal regrowth			P						Improved Barnes maze performance; increased motor behavioral index scores and beam balance test	**—**	Yoon et al., [101]
Asphyxial model of cardiac arrest			1 mA A- tDCS for 0.5 h with a constant direct current generator, repeated for four sessions with a resting interval of 1 h	MAP2, GAP-43, PSD-95, and SYN dramatically higher levels			P						Improves quantitative electroencephalogram; neurological deficit score and 96 h survival	**—**	Dai C et al., [118]

MCAO model	Acute+subacute	C-tDCS	15 min, once per day, 500 *μ*A administrated for 5 days in the acute and 5 days in the subacute phase, at a corresponding charge density of 128,571 C/m^2^ (higher than the one used in clinical trials)	Promoted neural stem cell differentiation to oligodendrocytes and neurons	P		P						Improved locomotor activity and athletic endurance deficits; accelerated recovery of limping gait	Inhibited Notch1 signaling pathway activation (DLL1 and Jagged1 downregulation and NUMB upregulation)	Zhang et al., [99]
	Acute		1/2 group-C-tDCS alternating 15 min on and 15 min off starting 45 min after MCAO and lasting 4 h. 1/2 group-same protocol but starting soon after MCAO and lasting 6 h. A constant current intensity of 0.2 mA (current density of 2.86 mA/cm^2^)	Reduced oxidative stress		P				P			Decreased number of spreading depolarizations; reduced infarct volume and area	Possibly C-tDCS blocks, the origin and the repeatedly spontaneous cycling of peri-infarction depolarizations	Notturno et al., [22]
		A-tDCS or/and C-tDCS	15 min at 500 *μ*A, starting 3 days after ischemia, for 10 days in total (A-tDCS or C-tDCS), with a pause of 2 days in the middle of sessions (5-2-5 days) and a charge density of 128,571 C/m^2^	Increased microglia polarization towards an M1 phenotype: iNOS-positive M1-polarized microglia	P		P	P					Accelerated functional recovery; only C-tDCS recruited oligodendrocyte precursors towards the lesion and supported M1-polarization of microglia	**—**	Braun et al., [113]
			20 min on–20 min off–20 min on of either C-tDCS or A-tDCS, starting after the first 30 min or at 4.5 hours after MCAO	C-tDCS, but not A-tDCS, reduced glutamate excitotoxicity		P		P			P	P	A-tDCS increased BBB permeability, but not C-tDCS; C-tDCS reduced the ischemic volume and brain edema; ameliorated functional deficits	Decrease of cortical glutamate synthesis and downregulation of NR2B NMDAR subunit	Peruzzotti-Jametti et al., [21]
			30 min daily, A-tDCS and C-tDCS 10 Hz, 0.1 mA, beginning 1 day after stroke for 3, 5, 7, 11, or 14 days	Reduced neuronal membrane permeability and ionic dysregulation			P						Early application of t-DCS from day 7 to day 14 after stroke may result in better motor function improvement than ultraearly intervention (within 3–5 days after stroke); also, it reduced the significantly increased hemichannel pannexin-1 mRNA expression on days 7 and 14	Ischemia may induce opening of the hemichannel pannexin-1 (protein family that forms large-pore nonselective channels in the plasma membrane of cells)	Jiang et al., [100]
4-vessel occlusion model			400 *μ*A constant current applied for 15 min, once, during cerebral ischemia	C-tDCS significantly decreased the levels of IL-1*β* and TNF-*α*, MDA, and NOS, while increasing the level of SOD; caused a significant decrease in NMDAR level, Bax and caspase-3 expressions, while increasing the Bcl-2 expression; significantly lower DNA fragmentation and neuronal death		P		P		P	P		Improved learning and memory dysfunctions	Antiapoptotic pathway Bcl-2	Kaviannejad et al., [167]

tDCS: transcranial direct current stimulation; C-tDCS: cathodal transcranial direct current stimulation; A-tDCS: anodal transcranial direct current stimulation; ES: electrical stimulation; mTOR: mammalian target of rapamycin; BDNF: brain-derived neurotrophic factor; GDNF: glial cell line-derived neurotrophic factor; VEGF: vascular endothelial growth factor; PI3K: phosphoinositide 3-kinase; MAP-2: microtubule-associated protein 2; GAP-43: growth-associated protein 43; PSD-95: postsynaptic density protein; SYN: synaptophysin; BBB: blood-brain barrier; DLL1: delta-like 1; MCAO: middle cerebral artery occlusion; iNOS: inducible nitric oxide synthase; NMDAR: N-methyl-D-aspartate receptor; s-tDCS: sham transcranial direct current stimulation; GABA: *γ*-aminobutyric acid; MDA: malondialdehyde; NOS: nitric oxide synthase; SOD: superoxide dismutase.

**Table 2:** Main clinical outcomes after transcranial electric stimulation.

Model	Stroke stage	Technique	Types of protocol	Reported results	Clinical outcome	Possible signalling pathway	Data
Clinical data	Healthy volunteers	A-tDCS and C-tDCS	Continuous currents for 4 s (excitability shifts during tDCS), 5 (short-lasting excitability shifts), 9 (C-tDCS), or 11 min (A-tDCS), with an intensity of 1.0 mA. A-tDCS was repeated 20 min after the first stimulation	A-tDCS can modulate GABAergic inhibition	Anodal stimulation enhances excitability, cathodal stimulation reduces it enhancement of neurotransmitter metabolism	Might be due to influences of remote cortical or subcortical structures	Nitsche et al., [103]
			A-tDCS 1 mA current, with a ramp up time of 10 s, held at 1 mA for 10 min, and then ramped down over 10 s. For sham stimulation, the current was ramped up over 10 s and then immediately switched off	A-tDCS caused locally reduced GABA, C-tDCS caused reduced glutamatergic neuronal activity with a highly correlated reduction in GABA	A-tDCS - decreased metabolism, C-tDCS - intensive neurotransmitter metabolism	Reduced activity of GAD-67, the rate-limiting enzyme in the major metabolic pathway for GABA synthesis	Stagg et al., [104]
	Acute	C-tDCS	A current of 1.5 mA or sham current delivered hourly for 20 min each, over a period of 6 hours and 20 min; C-tDCS started before completion of recanalization procedure in all patients	Reduced infarct volume of stroke patients receiving reperfusion therapy	Better motor improvement and more functional independence at 3 months post stroke; no major adverse effects (death or neurological deterioration); no statistical difference between the treated and sham groups	No data	Pruvost-Robieux et al., [107]
		C-tDCS, A-tDCS and bilateral tDCS	Each patient received 10 sessions (5 consecutive days for 2 weeks) of real or sham stimulation at 2 mA intensity and current density equivalent to 0.05 A/m^2^. For sham stimulation, the current was ramped up over 30 seconds and then turned off	No data	Significant motor recovery sustained at least three months beyond the intervention; decreased risk of falls; only in the bilateral stimulation group was reported an increase in the lower limb's motor skills		Andrade et al., [38]
		C-tDCS vs. A-tDCS	A current of 2 mA for 25 min daily for 6 consecutive days over the motor cortex hand area	A-tDCS over the affected hemisphere may be as effective as C-tDCS on the unaffected hemisphere to enhance recovery after acute ischemic stroke	Clinical improvements not only in the upper limb but also in the lower limb on the affected side		Khedr et al., [106]
	Chronic	A-tDCS	Current (1 mA) remained on for 20 min in the tDCS session and for up to 30 s in the sham session	The effect outlasted the stimulation period	Beneficial influence on skilled motor functions of the paretic hand in patients suffering from chronic stroke; significant functional improvement of the paretic hand compared with motor therapy alone		Hummel et al., [105]
			Applied with the anode positioned over the ipsilesional M1 and the cathode over the contralateral supraorbital region for 20 min (1 mA); sham current applied for only 1 min after which it was slowly tapered down to 0 for the remainder 19 min	Effects maintained 1 and 6 days after the completion of the training	No complications were reported; improved motor performance compared with motor practice or with practice combined with either intervention alone;		Celnik et al., [126]
		C-tDCS and A-tDCS	30 min of 1.5 mA direct current with the anode placed over the ipsilesional and the cathode over the contralesional motor cortex	Functional reorganization of the ipsilesional motor cortex	No adverse effects were observed; improved motor functions		Lindenberg et al., [36]
			Single session of 20 min with 1.5 mA current. The anode was over the M1 contralateral to the paretic limb and the cathode over the M1 contralateral to the nonparetic limb (current density 0.06 mA/cm^2^)	Effects maintained for 3 weeks	Improved retention of gains in motor function	Might be modulated through intracortical inhibitory pathways	Goodwill et al., [127]

A-tDCS: anodal transcranial direct current stimulation; C-tDCS: cathodal transcranial direct current stimulation; GABA: *γ*-aminobutyric acid; GAD-67: glutamate decarboxylase 67; tDCS: transcranial direct current stimulation.

**Table 3:** Main neuroprotective and neuroregenerative effects of transcranial magnetic stimulation.

Model	Stroke stage	Technique	Types of protocol	Results	Neurogenesis	Neuroprotection	Neural plasticity	Neuroinflammation	Angiogenesis	Excitability	Oxidative stress	BBB	Effects	Signalling pathway	Data
Healthy animals		HF-rTMS	Progressive number of trains (15 Hz, 5 s duration, 75 stimuli) separated by 10 s intervals (one treatment was delivered on day 1, up to five on day 5)	Augmented number of thin spines and enhanced dendritic complexity			✓						Did not increase anxiety in mice	Possible BDNF and calcium-dependent signaling pathways	Cambiaghi et al., [25]
Photothrombotic stroke model	Acute	cTBS	rTMS, 1/day, 5 min, days 1-6 after stroke, 3 pulses of 50 Hz, repeated every 200 ms, magnetic field adjusted to 200 gauss	Vascular repair and protection - RECA-1, protein collagen IV with FITC-labelled dextran leakage abated by over 50% in rTMS group rats; upregulation of IGFBP1, TGF*β*, VEGF, and PDGFR*β*; preserved neuronal morphology and synaptic structure; reduces microgliosis and induces a shift in in microglia phenotype; suppresses proinflammatory cytokine production;		✓	✓	✓	✓		✓	✓	Protected behavioral outcomes; attenuated infarct volume; promoted functional recovery	HIF-1*α* pathway; antiapoptotic pathways caspase-3 and caspase-9	Zong et al., [19]; Zong et al., [20]
MCAO model		HF-rTMS	Magnetic stimulation for 3 s followed by rest for 50 s and repeated 10 times (300 pulses per day) at a rate of 10 Hz, 3.5 T peak magnetic welds; performed at 24 h, 7 and 14 days after stroke	Increased number of BrdU and NESTIN+ cells; Bcl-2 expression was significantly lower and Bax expression was significantly higher; mRNAs of BDNF and TrkB levels were also significantly higher	✓	✓							Improvement of cognitive impairment	BDNF/TrkB signaling pathway and antiapoptotic pathways Bcl-2 and Bax	Guo et al., [146]
			A 3.5 T peak magnetic weld was applied to conscious rats at day 4 after cerebral ischemia, 10 sessions of stimulation over a 2-week period, seven times of five 1 s strains at a rate of 10 Hz with a 1 s intertrain interval per day (a total of 3,500 impulses)	Greater number of positive Bcl-2 cells and fewer Bax-positive cells in the rTMS group		✓							Beneficial effect on motor function on Barnes maze performance, motor behavioral index scores, and beam balance test	Antiapoptotic pathways Bcl-2 and Bax	Yoon et al., [145]
		HF -TMS and iTBS	20 Hz rTMS and iTBS, maximum stimulator output of the magnetic stimulator of 6.0 T, 7 and 14 days	Improvement of functional recovery, reduction of the infarcted area volume, neurogenesis (KI67, DCX, NESTIN)	✓								Elevated protein levels of BDNF and phosphorylated TrkB	BDNF/TrkB signaling pathway	Luo J et al., [148]
	Acute and chronic	LF-rTMS	2 times a day, 30 pulses each time, with a frequency of 0.5 Hz and magnetic field intensity of 1.33 tesla, when the animals were awake after MCAO, for 1, 7, 14, 21, and 28 days	Higher c-FOS expression, higher BDNF expression in 7, 14, and 21 days after stroke groups			✓						Improved functional outcome	BDNF/TrkB signaling pathway	Zhang et al., [26]
		LF-TMS and peripheral nerve stimulation	Paired associative stimulation with a frequency of 0.05 Hz 90 times over 4 weeks (TMS and left tibial nerve stimulation, 1/day); the interpair and interstimulus intervals were 20 seconds and 15 ms, respectively; 7, 14, and 28 days	Higher average BDNF and NMDAR1 expression levels		✓	✓			✓			Learning and memory amelioration, neuroplastic effect	BDNF/TrkB signaling pathway	Hu et al., [149, 150]

MCAO: middle cerebral artery occlusion; HF-rTMS: high-frequency repetitive transcranial magnetic stimulation; rTMS: repetitive transcranial magnetic stimulation; LF-rTMS: low-frequency repetitive transcranial magnetic stimulation; FITC: fluorescein isothiocyanate; IGFBP1: insulin-like growth factor binding protein 1; TGF*β*: transforming growth factor *β*; VEGF: vascular endothelial growth factor; PDGFR*β*: platelet-derived grow factor receptor beta; HIF-1*α*: hypoxia-inducible factor 1*α*; BDNF: brain-derived neurotrophic factor; TrkB: tropomyosin receptor kinase B; iTBS: intermittent theta burst stimulation; cTBS: continuous theta burst stimulation; NMDAR1: N-methyl-D-aspartate receptor 1.

**Table 4:** Main clinical outcomes after transcranial magnetic stimulation.

Model	Stroke stage	Technique	Types of protocol	Reported results	Clinical outcome	Signalling pathway	Data
Clinical data	Healthy volunteers	LF-rTMS	1 Hz rTMS for 20–22 min at an intensity of 90% RMT (1 Hz rTMS: train of 10 pulses, 1 s wait time between trains, 120 trains, total pulses = 1200; 5 Hz rTMS: train of 25 pulses, 45 s wait time between trains, 24 trains, total pulses = 600); one volunteer additionally received 5 Hz rTMS in a separate session, 3 weeks after the 1 Hz protocol	Modulates neurotransmitter metabolism (increased GABA concentrations)	No significant changes for functional connectivity	No data	Gröhn et al., [143]
	Acute and subacute	LF-rTMS	20 minutes with 1 Hz rTMS, 5 days per week for a 2-week period	No side effects	Motor improvement and cognitive functions amelioration (unilateral spatial neglect and aphasia)	No data	Zheng et al., [152] Cha and Kim, [42] Weiduschat et al., [154]
			For 20-30 min each time, 1 time/day, and 5 times/week, 4 weeks	Higher SOD levels, lower MDA and ET-1	Improvement in cerebral oxygen metabolism and regulation of brain neurotransmitter	No data	Peng et al., [141]
		c-TBS	In every session, 3-pulse bursts at 50 Hz repeated every 200 msec for 40 s were delivered at 80% of the active motor threshold over the left PPC (600 pulses). 15 every day 2 sessions of left PPC cTBS were applied with an interval of 15 minutes. Stimulation lasted for 10 days (5 days per week, Monday to Friday) and was applied daily at the same hour every morning (11 AM) to all patients	Possibly by counteracting the hyperexcitability of left hemisphere parieto-frontal circuits	Recovery from visual spatial neglect	No data	Koch et al., [155]
		HF-rTMS	rTMS (daily at noon) consisted of ten 10-second trains of 3 Hz stimulation with 50 seconds between each train, for 10 days	No side effects	10 consecutive days of rTMS employed as an add-on intervention to normal physical and drug therapies improved immediate clinical outcome in early stroke patients	No data	Khedr et al., [39]
			rTMS applied for 10 min every day for 5 consecutive days, each session consisting of 10 trains of 3 Hz stimulation for 10 s and then repeated every minute	Increased excitability of the corticobulbar projections from both hemispheres with better projection from the stroke hemisphere	Motor improvement and recovery from dysphagia (maintained for 2 months)	No data	Khedr et al., [40]
			A daily dose of 1000 pulses of subthreshold 10 Hz rTMS, 10 days	Higher movement accuracy; variable benefits in motor performance	Possible variable functional integrity of the corticospinal tract and different BDNF genotype	No data	Chang et al., [43]; Chang et al., [168]
		iTBS and LF-rTMS	7 days after stroke, for 10 days, iTBS (600 pulses) to the affected hemisphere; 1 Hz stimulation (1200 pulses) of the unaffected motor cortex hand area, also 10 days	No complications; motor improvement by iTBS; spasticity reduction by contralesional 1 Hz stimulation	Enhance motor recovery	No data	Watanabe et al., [41]
	Chronic	LF-rTMS	1 Hz, 25 minutes, a subthreshold rTMS over the unaffected hemisphere	Increase in the excitability of the affected motor cortex	rTMS improved the motor learning of the affected hand in patients after stroke; enhanced motor skill acquisition and training effect	No data	Takeuchi et al., [44]
		HF-rTMS	Pulses were applied twice daily at 3 Hz for 10 s with a 25-second interval, 20 times per session, alternating between left and right hemispheres (300 pulses for the left hemisphere and 300 pulses for the right hemisphere in one treatment session, 1,200 pulses per day) and were followed by 20 min of intensive swallowing rehabilitation exercise	No deterioration of neurological symptoms or adverse reactions such as convulsions or pneumonia	Improved laryngeal elevation delay time	No data	Momosaki et al., [159]
			For the bilateral stimulation group, 500 pulses of 10 Hz rTMS over the ipsilesional and 500 pulses of 10 Hz rTMS over the contralesional motor cortices over the cortical areas that project to the mylohyoid muscles were administered daily, 2 consecutive weeks. For the unilateral stimulation group, 500 pulses of 10 Hz rTMS over the ipsilesional motor cortex over the cortical representation of the mylohyoid muscle and the same amount of sham rTMS over the contralesional hemisphere were applied	Magnetic stimulation over the cortical areas projecting to the mylohyoid muscles is effective as an additional treatment strategy to traditional dysphagia therapies	Swallowing parameters showed an improvement in the bilateral simulation group	No data	Park et al., [169]
		LF-rTMS and HF-rTMS	1 Hz rTMS over the unaffected hemisphere, 10 Hz rTMS over the affected hemisphere or bilateral rTMS comprising both the 1 Hz and 10 Hz rTMS	No side effects	An improvement in the motor function of the paretic hand	No data	Takeuchi et al., [156]
		iTBS	Bursts of three pulses at 50 Hz given every 200 milliseconds in two-second trains, repeated every 10 seconds over 200 seconds for a total of 600 pulses	No side effects	Improvements in semantic fluency (language skills), stronger language lateralization to the dominant left hemisphere	No data	Szaflarski et al., [160]

LF-rTMS: low-frequency repetitive transcranial magnetic stimulation; rTMS: repetitive transcranial magnetic stimulation; RMT: resting motor threshold; GABA: *γ*-aminobutyric acid; PPC: posterior parietal cortex; cTBS: continuous theta burst stimulation; BDNF: brain-derived neurotrophic factor; SOD: superoxide dismutase; MDA: malondialdehyde; ET-1: endothelin-1; iTBS: intermittent theta burst stimulation.

## Data Availability

Data are available on reasonable request.
